# Release of glutamate and CGRP from trigeminal ganglion neurons: Role of calcium channels and 5-HT_1 _receptor signaling

**DOI:** 10.1186/1744-8069-4-12

**Published:** 2008-04-16

**Authors:** Yan Xiao, Judith A Richter, Joyce H Hurley

**Affiliations:** 1 Department of Biochemistry and Molecular Biology, Stark Neurosciences Research Institute, Indiana University School of Medicine, 950 West Walnut Street, Indianapolis, Indiana, 46202, USA; 2 Department of Pharmacology and Toxicology, Indiana University School of Medicine, 635 Barnhill Drive, Indianapolis, Indiana, 46202, USA

## Abstract

**Background:**

The aberrant release of the neurotransmitters, glutamate and calcitonin-gene related peptide (CGRP), from trigeminal neurons has been implicated in migraine. The voltage-gated P/Q-type calcium channel has a critical role in controlling neurotransmitter release and has been linked to Familial Hemiplegic Migraine. Therefore, we examined the importance of voltage-dependent calcium channels in controlling release of glutamate and CGRP from trigeminal ganglion neurons isolated from male and female rats and grown in culture. Serotonergic pathways are likely involved in migraine, as triptans, a class of 5-HT_1 _receptor agonists, are effective in the treatment of migraine and their effectiveness may be due to inhibiting neurotransmitter release from trigeminal neurons. We also studied the effect of serotonin receptor activation on release of glutamate and CGRP from trigeminal neurons grown in culture.

**Results:**

P/Q-, N- and L-type channels each mediate a significant fraction of potassium-stimulated release of glutamate and CGRP. We determined that 5-HT significantly inhibits potassium-stimulated release of both glutamate and CGRP. Serotonergic inhibition of both CGRP and glutamate release can be blocked by pertussis toxin and NAS-181, a 5-HT_1B/1D _antagonist. Stimulated release of CGRP is unaffected by Y-25130, a 5-HT_3 _antagonist and SB 200646, a 5-HT_2B/2C _antagonist.

**Conclusion:**

These data suggest that release of both glutamate and CGRP from trigeminal neurons is controlled by calcium channels and modulated by 5-HT signaling in a pertussis-toxin dependent manner and probably via 5-HT_1 _receptor signaling. This is the first characterization of glutamate release from trigeminal neurons grown in culture.

## Background

Several lines of evidence indicate that both glutamate and CGRP are important neurotransmitters in migraine. For example, clinical studies indicate that CGRP is elevated in plasma or platelets during migraine episodes [1] (however, see Tvedskov *et al*., 2005 [2]), presumably due to activation of the trigeminovascular system. CGRP antagonists are being tested with some success as therapeutic agents for acute migraine [3,4]. In a manner analogous to CGRP, most studies indicate that glutamate is elevated in the cerebrospinal fluid, plasma and/or platelets of migraine sufferers [5-7]. Likewise, a novel AMPA/GluR5 antagonist has recently been shown to be efficacious in migraine [8]. Understanding the mechanisms which control the release of these two neurotransmitters may help in the treatment of migraine as well as other painful diseases associated with the trigeminal nerve including cluster headache.

The regulated release of neurotransmitters, such as glutamate and CGRP, is controlled by voltage-dependent calcium channels, including P/Q- type channels. Missense mutations in the P/Q-type channel have been identified in Familial Hemiplegic Migraine (FHM), a type of migraine with severe aura symptoms. The FHM mutation (R192Q) has gain-of-function effects in cell lines (for review, see Pietrobon, 2003 [9]) and results in increased activity and excess release of neurotransmitter in a knock-in mouse [10]. Functional studies to characterize the normal role of the P/Q-type channel in trigeminal ganglion neurons may help in further understanding the pathophysiology of migraine.

Currently, the most widely used specific acute anti-migraine treatments are a class of 5-HT_1B/1D _receptor agonists, the triptans, and clinical studies have demonstrated that CGRP levels return to basal after sumatriptan and in a similar time frame as headache relief [11]. Their mechanism of action is not completely understood, but one hypothesis suggests that triptans inhibit neurotransmitter release from trigeminal neurons and subsequently attenuate vasodilation, neurogenic inflammation and central sensitization. As both glutamate and CGRP are co-localized with 5-HT_1B_, 5-HT_1D _and/or 5-HT_1F _receptors in trigeminal neurons [12,13] it was suggested that the release of glutamate may be regulated in a similar manner by 5-HT_1 _receptor activation. Pre-clinical studies have provided indirect evidence that triptans can inhibit the release of glutamate or attenuate the action's of glutamate in spinal cord slices presumably by a pre-synaptic mechanism [14,15]. However, previous studies have not directly examined the regulation of glutamate release by calcium channels and 5-HT signaling in trigeminal neurons grown in culture. Conducting our experiments on cells grown in culture allows the delivery of drugs at known concentrations and without the presence of additional modulatory pathways. Our goals in these studies are two-fold: 1) to characterize the roles of the P/Q-, N- and L-type calcium channels in release of glutamate and CGRP from trigeminal neurons grown in culture and 2) to examine the effects of 5-HT receptor activation on release of glutamate and CGRP.

## Results

### Role of calcium channels in release of glutamate and CGRP from trigeminal neurons

We examined the role of voltage-dependent calcium channels in release of glutamate and CGRP from trigeminal neurons isolated from male rats or female rats grown in culture. Several measures were used to ensure that comparable numbers of viable cells were present in all wells within a dish. Cells in representative wells were counted immediately before the release experiment and trypan blue exclusion was used to assess the viability of cells before and after the release experiment. Cultures contained 1800–2500 viable cells/well from male or female rats at the time the release studies were performed. In addition, the average basal releases of neurotransmitter from each treatment group (in the absence of drugs) in an experiment were within 30% of each other. The cells exhibited robust KCl-stimulated neurotransmitter release at least four-fold over the basal levels. Post-treatment basal release (in the absence of drugs) was measured in all CGRP assays and some glutamate assays and was not more than 50% higher than initial basal levels indicating that the release assay did not damage the cells or induce cell lysis (data not shown). These observations when taken together indicate that similar numbers of viable cells were present in all wells within an experiment. We also assessed the between experiment variability. For example, the basal CGRP values for 32 independent experiments from male rats averaged 15 ± 1.3 fmol/well/10 min and ranged from 5 – 40 fmol/well/10 min. In these 32 experiments the values for stimulated CGRP release averaged 115 ± 4.5 fmol/well/10 min and ranged from 80 – 175 fmol/well/10 min. The values for basal and stimulated release of CGRP did not differ significantly in trigeminal neurons from female rats nor did the effects of calcium channel blockers of 5-HT therefore the data from male and female rats was pooled except where indicated.

**Figure 1:**
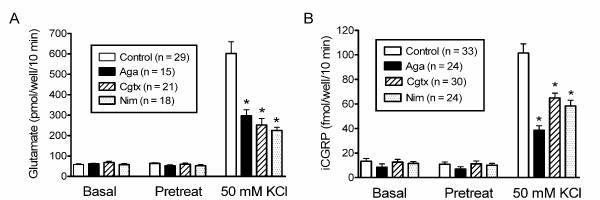
**P/Q-, N- and L-type calcium channels contribute to glutamate and CGRP release from trigeminal neurons grown in culture**. Panels A and B depict glutamate or immunoreactive CGRP in the buffer measured after successive 10 minute incubations in the absence and presence of 50 mM KCl and/or drugs. Drugs were present as indicated during "Pretreat" and "50 mM KCl" incubations but not during the "Basal" incubation. Data is presented as the mean ± S.E.M. and the number of wells tested is indicated in parentheses. An asterisk indicates a significant difference (p < 0.05) between the transmitter release from control cells and from cells treated with calcium channel blockers. Potassium-stimulated release of glutamate was inhibited by 1 μM ω-Aga TK, 1 μM ω-Cgtx GVIA and 1 μM nimodipine while basal glutamate release was not altered (Panel A). Potassium-stimulated release of CGRP was inhibited by 1 μM ω-Aga TK, 1 μM ω-Cgtx GVIA and 1 μM nimodipine while basal CGRP release was not altered (Panel B).

In the release experiments 50 mM KCl was used to stimulate release of transmitter from cultured cells. Cultures were incubated for successive 10 minute intervals under basal and stimulating conditions and after each 10 minute interval, the buffer was exchanged and glutamate and/or CGRP was assayed. Various subtype-specific calcium channel blockers were added to the release buffer under non-stimulating (pretreatment) and stimulating conditions (50 mM KCl). The release obtained in the absence of a blocker was compared to the release in the presence of a blocker to determine the contribution of each of the channel types to CGRP release. As seen in Fig. 1A, exposing trigeminal ganglion neurons from male rats to 50 mM KCl stimulated glutamate release by more than ten-fold from a basal level of 58 ± 4 pmol/well/10 min to 602 ± 57 pmol/well/10 min. The addition of 1 μM ω-Aga TK, a P/Q-type channel blocker [16] decreased the magnitude of the potassium-stimulated glutamate release by 51% to 297 ± 29 pmol/well/10 min compared to potassium-stimulated glutamate release in the absence of blocker (p < 0.05), whereas 200 nM ω-Aga TK decreased the potassium-stimulated release by 37 ± 10% (n = 12, p < 0.05, data not shown). ω-Cgtx (1 μM), an N-type channel blocker [17] decreased the magnitude of the potassium-stimulated glutamate release by 58% to 252 ± 32 pmol/well/10 min compared to potassium-stimulated glutamate release in the absence of blocker (p < 0.05). Nimodipine (1 μM), an L-type channel blocker [18] decreased the magnitude of the potassium-stimulated glutamate release by 63% to 225 ± 16 pmol/well/10 min compared to potassium-stimulated glutamate release in the absence of blocker (p < 0.05). None of the three calcium channel blockers reduced the basal release of glutamate. The results of these experiments suggest that P/Q-, N- and L-type channels each mediate a significant fraction of depolarization-associated glutamate release from cultured trigeminal neurons.

We also determined the importance of calcium channels in potassium-stimulated release of CGRP from trigeminal neurons from male and female rats (Fig. 1B). Potassium stimulated release of CGRP by over seven-fold from a basal level of 13 ± 2 fmol/well/10 min to 101 ± 8 fmol/well/10 min. ω-Aga TK (1 μM) decreased the magnitude of the potassium-stimulated CGRP release by 61% to 39 ± 4 fmol/well/10 min compared to potassium-stimulated CGRP release in the absence of blocker (p < 0.05). In additional experiments, 200 nM ω-Aga TK inhibited potassium-stimulated release of CGRP by 15 ± 4% (n = 12, data not shown). The addition of ω-Cgtx (1 μM) inhibited potassium-stimulated CGRP release by 36% to 65 ± 4 fmol/well/10 min compared to potassium-stimulated CGRP release in the absence of blocker (p < 0.05). Nimodipine (1 μM) decreased the magnitude of the potassium-stimulated release of CGRP by 43% to 58 ± 5 fmol/well/10 min compared to potassium-stimulated CGRP release in the absence of blocker (p < 0.05). None of the calcium channel blockers reduced the basal release of CGRP.

### Regulation of glutamate and CGRP release by 5-HT receptors

**Figure 2:**
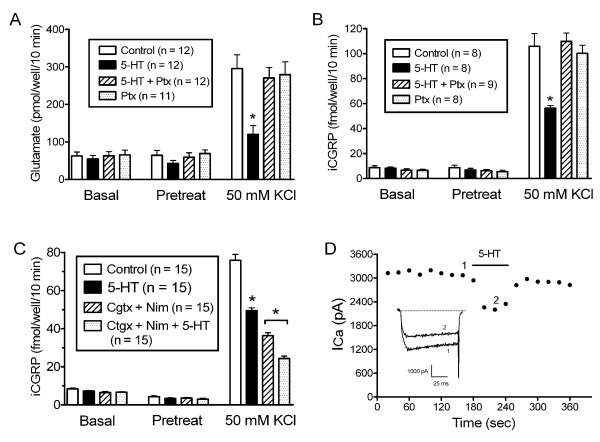
**Serotonin inhibits release of glutamate and CGRP from trigeminal neurons in a pertussis-toxin sensitive manner**. Panels A – C illustrate glutamate or immunoreactive CGRP in the buffer measured after successive 10 minute incubations in the absence and presence of 50 mM KCl and/or drugs. Drugs were present as indicated during "Pretreat" and "50 mM KCl" incubations but not during the "Basal" incubation. Data is presented as the mean ± S.E.M. and the number of wells tested is indicated in parentheses. An asterisk indicates a significant difference (p < 0.05) between the transmitter release from control cells and from cells treated with serotonin and/or pertussis toxin. Serotonin (10 μM) inhibits KCl-stimulated but not basal release of glutamate (Panel A). Overnight pertussis toxin treatment (100 ng/ml) blocks the serotonergic inhibition of KCl-stimulated glutamate release without affecting basal or KCl-stimulated release. Serotonin (10 μM) inhibits KCl-stimulated but not basal release of CGRP (Panel B). Overnight pertussis toxin treatment (100 ng/ml) blocks the serotonergic inhibition of KCl-stimulated CGRP release without affecting basal or KCl-stimulated release from trigeminal neurons. Serotonin inhibits KCl-stimulated release of CGRP from trigeminal neurons from female rats in the absence and presence of the calcium channel blockers, 1 μM ω-Cgtx GVIA and 1 μM nimodine to a similar extent (Panel C). Serotonin (1 μM) reversibly inhibits calcium current amplitude (Panel D). Peak currents were plotted against time for this cell. The trigeminal neurons were depolarized to 0 mV for 100 msec every 20 seconds from a holding potential of -80 mV. The horizontal bar indicates perfusion of 1 μM 5-HT. Inset, superimposed current traces from the indicated time points.

We examined the effects of 5-HT on potassium-stimulated release of glutamate and CGRP. Fig. 2A illustrates the effects of 10 μM 5-HT on basal and potassium-stimulated release of glutamate from male rat trigeminal neurons grown in culture. Exposing trigeminal neurons to 50 mM KCl increased glutamate release by greater than four-fold from a basal level of 63 ± 11 pmol/well/10 min to 296 ± 37 pmol/well/10 min. The addition of 10 μM 5-HT significantly reduced potassium-stimulated release of glutamate by 59% to 120 ± 24 pmol/10 min/well (p < 0.05), without affecting basal release. Likewise, 1 μM 5-HT significantly reduced the potassium-stimulated release of glutamate by 35 ± 9% (n = 12, data not shown). Overnight treatment of the cultures with pertussis toxin (100 ng/ml) abolished the effect of 10 μM 5-HT, indicating the involvement of Gα_i_/Gα_o _signaling. Pertussis toxin did not affect basal or stimulated release.

As shown in Fig. 2B, 5-HT inhibited release of CGRP from trigeminal neurons from male and female rats grown in culture. Potassium stimulated the release of CGRP by almost twelve-fold from a basal level of 9 ± 1 fmol/well/10 min to 106 ± 10 fmol/well/10 min. The addition of 10 μM 5-HT reduced potassium-stimulated release of CGRP by 47% to 56 ± 2 fmol/10 min/well (p < 0.05), without affecting basal release. Potassium-stimulated release of CGRP was not significantly decreased by 1 μM 5-HT (13 ± 4% (n = 12), data not shown). Overnight treatment of the cultures with pertussis toxin (100 ng/ml) blocked the effect of 10 μM 5-HT without affecting basal or stimulated release of CGRP.

As we are particularly interested in the P/Q-type channel we compared the effects of 5-HT on the release of CGRP from trigeminal cultures from female rats in the presence and absence of N- and L-type channel activity (Fig. 2C). When no channel blockers were present potassium stimulated release of CGRP by almost ten-fold from 8.35 ± 0.48 to 75.8 ± 3.13 fmol/well/10 min and 10 μM 5-HT inhibited the stimulated release by 35% (p < 0.05). In the presence of 1 μM nimodipine and 1 μM ω-Cgtx GVIA, the stimulated release was 36.4 ± 1.5 fmol/well/10 min (or 48% of the stimulated release in the absence of these two blockers). The addition of 10 μM 5-HT inhibited the nimodipine- and ω-Cgtx GVIA-insensitive release by 33% (p < 0.05).

We hypothesize that 5-HT receptor activation may inhibit calcium channel function and subsequently reduce release of neurotransmitters. We have used patch clamp electrophysiology to characterize the acute effects of 5-HT on calcium currents in trigeminal neurons [19]. In the example in Fig. 2D, 1 μM 5-HT reversibly inhibited peak calcium current amplitude by 31% in a small capsaicin-sensitive trigeminal neuron, suggesting a mechanism where 5-HT may reduce release of CGRP.

**Figure 3:**
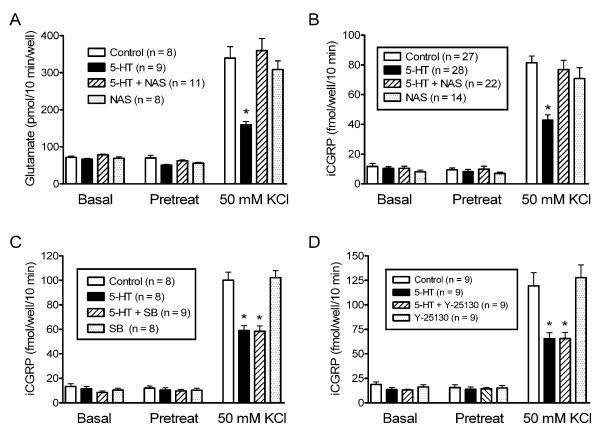
**A 5-HT_1 _receptor antagonist but not 5HT_2 _or 5HT_3 _antagonists block serotonergic inhibition of neurotransmitter release from trigeminal neurons**. Panels A-D illustrate glutamate or immunoreactive CGRP in the buffer measured after successive 10 minute incubations in the absence and presence of 50 mM KCl and/or drugs. Drugs were present as indicated during "Pretreat" and "50 mM KCl" incubations but not during the "Basal" incubation. Data is presented as the mean ± S.E.M. and the number of wells tested is indicated in parentheses. An asterisk indicates a significant difference (p < 0.05) between the transmitter release from control cells and from cells treated with serotonin and/or serotonin receptor antagonists. NAS-181 (1 μM), a 5-HT_1 _receptor antagonist, blocks the serotonergic inhibition of release of glutamate and CGRP (Panels A and B, respectively) without affecting basal or stimulated release. SB 200646 (1 μM), a 5-HT_2B/2C _antagonist does not block 5-HT (10 μM) inhibition of KCl-stimulated release of CGRP (Panel C) or alter basal or KCl-stimulated release. Y-25130 (500 nM), a 5-HT_3 _antagonist does not block 5-HT (10 μM) inhibition of KCl-stimulated release of CGRP (Panel D) or alter basal or KCl-stimulated release.

Sensory neurons express several families of 5-HT receptors including 5-HT_1_, 5-HT_2 _and 5-HT_3 _[20-23]. Therefore, we used subtype selective antagonists to determine the roles of 5-HT_1_, 5-HT_2 _and 5-HT_3 _receptors in controlling neurotransmitter release. As depicted in Fig. 3A, 5-HT significantly reduced stimulated release of glutamate by 53% while NAS-181, a 5-HT_1B/1D _receptor antagonist [24], blocked the inhibitory effect of 5-HT without affecting basal or stimulated release. Likewise, NAS-181 blocked the ability of 5-HT to inhibit potassium stimulated release of CGRP (Fig. 3B) without affecting basal or stimulated release. In contrast, neither SB 200646, a 5-HT_2B/2C _antagonist [25] or Y-25130, a 5-HT_3 _antagonist [26] was able to block the inhibitory actions of 5-HT on CGRP release (Figs. 3C and 3D). In summary, 5-HT inhibits glutamate and CGRP release from trigeminal cultures by pertussis-toxin sensitive signaling pathways, probably via 5-HT_1 _receptors.

## Discussion

To our knowledge this is the first assessment of the regulation of glutamate and CGRP release from adult rat trigeminal ganglion neurons grown in culture. Several lines of evidence suggest that glutamate is important in nociceptive signaling in the trigeminal ganglion. NMDA, kainate, AMPA and metabotropic glutamate receptors are present in the superficial lamina of the trigeminal nucleus caudalis in the rat [27]. Also, NMDA and AMPA receptor antagonists block transmission of trigeminovascular nociceptive signals [28,29] and attenuate the elevated c-*fos *levels seen in trigeminal nucleus caudalis after intracisternal capsaicin injection [30]. Our data demonstrate that P/Q-type channels as well as N- and L-type channels each contribute significantly to glutamate release from cultured trigeminal cells from male rats in neurotransmitter release assays. In addition, 5-HT inhibits glutamate release and this inhibition is blocked by a 5-HT_1 _receptor antagonist and is pertussis-toxin sensitive. Our data suggests that 5-HT receptor signaling regulates the release of glutamate from trigeminal neurons.

Our data demonstrates that P/Q-type, N- and L-type channels each contribute significantly to CGRP release from cultured trigeminal cells from both male and female rats. While several studies have examined the regulation of CGRP release from trigeminal neurons by triptans [31], adenosine [32] and capsaicin [33] none have focused on identifying which calcium channels are coupled to the release of CGRP. Some previous studies have determined that the P/Q-type channel may not be important for release of CGRP from dorsal root ganglion [34] while others suggest that the P/Q-type channel is important in spinal nociceptive processes [35] and particularly in inflamed tissue [36] in the spinal cord. Our data indicates that P/Q-, N- and L-type calcium channels are coupled to release of CGRP from trigeminal neurons. However, it is unclear at this time whether tissue specific differences exist in the coupling of calcium channels to neurotransmitter release in trigeminal ganglion and dorsal root ganglion.

It has been hypothesized that sumatriptan blocks migraine pain by attenuating the elevated release of CGRP from trigeminal nerves. The seminal work of Durham and Russo [31,37] had previously demonstrated that sumatriptan can inhibit CGRP promoter activity and stimulated release of CGRP from trigeminal neurons from neonatal rats. In this study and our previous work [19] we demonstrate that 5-HT, probably via a 5-HT_1 _receptor pathway, inhibits stimulated CGRP release from cultured adult rat trigeminal neurons. Signaling through 5-HT_1 _receptors is usually via activation of heterotrimeric G proteins of the Gα_i_/Gα_o _class which couple to inhibition of adenylate cyclase [38]. Our results are consistent with the involvement of Gα_i_/Gα_o _proteins in 5-HT's signaling pathway as pertussis toxin abolishes the actions of 5-HT on release of both neurotransmitters. However, in earlier studies in neonatal rat trigeminal neuronsor cell lines [31,37] sumatriptan increased intracellular calcium rather than inhibited adenylate cyclase. Pertussis toxin did not block sumatriptan induced increases in intracellular calcium in CA77 cells, a neuronal-like cell line. In addition, Carruthers *et al*., (2001) [32] observed that sumatriptan did not inhibit forskolin stimulated release of CGRP from rat trigeminal neurons. At present it is not certain whether these interesting differences can be attributed to the use of neonatal versus adult rats, cell lines, different agonists or other methodological differences. In consideration of these differences and the clinical importance of the triptans, additional studies in our adult rat trigeminal cell cultures with triptans are warranted.

As the prevalence of migraine and other painful disorders associated with the trigeminal system is higher in females, we have compared the regulation of neurotransmitter release in trigeminal neurons from male and female rats grown in culture. Similar to our results in trigeminal neurons from male rats, P/Q-, N- and L-type channels all contribute to release of CGRP in female rats. The effects of 5-HT on release of CGRP from trigeminal neurons are not different in male and female rats either. These results suggest that the serotonergic regulation of CGRP release from trigeminal neurons grown in identical culture conditions is similar in male and female rats, leading us to pool data from male and female rats in this report. Notwithstanding the similarity in regulation of CGRP release we observed and the difficulties of doing these kinds of comparisons in isolated cells, previous work indicates that sex differences are present in signaling in the trigeminal pathway. A compelling body of evidence from Bereiter's group [39-41] indicates that neurotransmitter release and pain processing differ in male and female rats and during the estrus cycle in the trigeminal system.

As P/Q-, N- and L-type channels all contribute to neurotransmitter release in our assays and all three channel types are known to be highly regulated by G protein coupled receptors we have compared the effects of 5-HT on release of CGRP in the absence and presence of N- and L-type channel blockers (Fig. 2C). Under these conditions the N- and L-type channel activity will be blocked and therefore the predominant channels likely to be contributing to release are the P/Q-type and perhaps R-type channels, as T-type channels will be inactive during the long KCl depolarizing stimulus, i.e., T-type currents are maximally activated at lower voltages and almost completely inactive in the presence of 50 mM KCL. The effects of 5-HT in the absence and presence of N- and L-type channel activity are very similar (35 vs. 33%, respectively), suggesting that the P/Q-type channel may be the primary target of 5-HT activity. We have also demonstrated that 5-HT inhibits calcium current amplitude in trigeminal neurons (Fig 2D and [19]). The data in this report and our previous studies are in agreement with the hypothesis that serotonergic inhibition of neurotransmitter release in trigeminal neurons may be due to 5-HT effects on calcium channel function_._

While P/Q-, N- and/or L-type calcium channels are known to be critical for neurotransmitter release from many central and peripheral nerve endings, their role in neurotransmitter release from trigeminal neurons had not been previously established. In this study, we examined the role of P/Q-, N- and L-type calcium channels in potassium-stimulated release of glutamate and CGRP from cultured trigeminal neurons. Although our data indicates that P/Q-, N- and L-type channels each mediate a high degree of neurotransmitter release in cultured trigeminal cells, we cannot rank the relative importance of each channel type as the relative contribution of voltage-dependent calcium channels in neurotransmitter release is dependent on the conditions used to stimulate release [42]. For example, the long duration of the depolarizing potassium stimulus may increase the apparent contribution of the L-type channel to CGRP release, due to the slow Ca^2+^-dependent inactivation of L-type channels compared to the faster voltage-dependent inactivation exhibited by the P/Q- and N-type channels (but see Lipscombe *et al*, 2004 [43]). In addition, this assay in neurons grown in culture does not differentiate release from soma and release from terminals. Release of neurotransmitter from the soma of sensory neurons has been described [33,44,45] but the functional significance is not well-understood. It has been suggested that somal release from sensory ganglion may mediate intraganglionic transmission [33,46] and have a central role in excitability. Somal release of transmitters such as ATP from neurons within sensory ganglion may be an important form of communication between neurons and glia [47,48] Our electrophysiology data reveals that all 3 channel types are localized to the cell soma [19] and therefore could theoretically mediate neurotransmitter release from the soma of trigeminal ganglion neurons.

Our study which directly assesses the role of calcium channels in neurotransmitter release from cultured trigeminal neurons is in agreement with several *in vitro *and *in vivo *studies. For instance, voltage-dependent calcium channels, including P/Q-type calcium channels, are localized to trigeminal presynaptic nerve terminals in the dura and the trigeminal nucleus caudalis where they have a prominent role in trigeminovascular nociception [49-53]. The P/Q-type calcium channel modulates trigeminal nociception indirectly through its role in descending pain pathways such as periaqueductal gray [54] as well. P/Q-type channels may be involved in initiation of migraine attacks and/or aura symptoms as well as nociceptive transmission. In addition, release of glutamate in the cortex, which may trigger cortical spreading depression (sometimes manifest as aura symptoms) and/or migraine pain by activating the trigeminovascular system, is controlled predominantly by P/Q-type channels (for review, see Moskowitz, 2004 [55]). Overall, our data when taken together with previous studies is consistent with an important, though not exclusive, role for the P/Q-type channel in release of neurotransmitters from trigeminal neurons.

## Conclusion

In summary, our data indicates that P/Q-, N- and L-type calcium channels are important in release of glutamate and CGRP from trigeminal neurons. In addition, our data demonstrates that 5-HT, most likely through 5-HT_1 _receptors, can inhibit release of both neurotransmitters to a similar degree. To our knowledge this is the first report demonstrating the regulation of glutamate release from trigeminal neurons and future studies will allow us to examine the mechanisms controlling release more thoroughly.

## Methods

### Chemicals

Stock solutions of nimodipine and SB 200646 hydrochloride (Tocris, Ellisville, MO, USA) were dissolved in DMSO, diluted daily in release buffer and protected from light. Stock solutions of ω-Agatoxin TK (Alomone Laboratories, Jerusalem, Israel), ω-Conotoxin GVIA (Alomone Laboratories), 5-HT, NAS-181 (Tocris) and Y-25130 hydrochloride (Tocris) were prepared in H_2_O and were diluted daily into release buffer. Pertussis toxin (Calbiochem, San Diego, CA, USA) was prepared in H_2_0 and diluted into F12 growth medium. CGRP peptide was from Tocris and CGRP antiserum was kindly provided by Dr. Michael Iadorola (NIH, Bethesda, MD). Other reagents were obtained from Sigma Chemical Co. (St. Louis, MO) unless indicated.

### Cell culture

All animal procedures were approved by the Animal Care and Use Committee at the Indiana University School of Medicine. Trigeminal ganglia neurons were isolated and grown in culture by a modification of the methods of Eckert *et al*. (1997) [56]. Briefly, male (100 – 150 g) or age-matched female Sprague-Dawley rats were rendered unconscious with carbon dioxide before cervical dislocation. For each experiment, trigeminal ganglia from 4–9 male female rats were dissected from the bony base of the brain, rinsed in ice-cold Puck's saline (Ca^2+ ^free, Mg^2+ ^free) and minced. All tissue was pooled and single cells were isolated by sequential exposure to Puck's saline containing papain (20 units/ml, Worthington, Lakewood, NJ, USA) followed by collagenase (0.1%) and dispase (0.25%, Roche, Indianapolis, IN, USA) for 18–20 minutes each. After washing, cells were resuspended in F12 medium (Gibco, Carlsbad, CA, USA) containing 10% fetal calf serum (Gibco), 2 mM glutamine, 100 ug/ml penicillin and streptomycin and 30 ng/ml NGF (Harlan Bioproducts, Indianapolis, IN, USA) and maintained at 37°C and 5% CO_2_. The contribution of non-neuronal cells was reduced by adding 50 μM 5-fluoro-2'-deoxyuridine and 150 μM uridine to the media. Cells were plated onto 12 well plates coated with polylysine and laminin. The F12 growth medium was changed every second day and the cells were used for release studies 7 – 10 days after plating.

### Electrophysiology studies

Trigeminal neurons were isolated as described above and plated on glass coverslips coated with polylysine and laminin. Whole cell patch clamp recordings were conducted 1–2 days after plating. Gigaohm seals were obtained and calcium currents were measured in TEA/Ba based extracellular recording solution and electrode resistance was 0.8 – 1.5 MΩ when filled with a CsCl-based internal solution [57]. Membrane currents were recorded on a computer using PCLAMP software (Axon Instruments, Union City, CA). Ionic currents were elicited by step depolarizations of 100 ms duration to a test potential of 0 mV from a holding potential of -80 mV every 20 seconds. Leak and capacitive currents were subtracted on-line with a P/4 protocol. Series resistance was compensated up to 70–80%. Capsaicin sensitivity was tested after calcium current recording by perfusing 1 μM capsaicin at a holding potential of -70 mV.

### Release studies

Briefly, the protocol for release studies is as follows [58]. All solutions and cells were maintained at 37°C during the release experiments. Prior to the release experiment, cells were washed three times with HEPES buffer containing 25 mM HEPES, 140 mM NaCl, 3.5 mM KCl, 2.5 mM CaCl_2_, 1 mM MgCl_2_, 3.3 mM D-glucose, and 1% bovine serum albumin (pH 7.4). The cells were then incubated with HEPES buffer at 37°C for 1 h followed by three more washes. Cells were then exposed to successive 10 minute incubations in the absence or presence of drugs to assay release. For example, basal or resting immunoreactive CGRP release was measured in all wells after 10 minute incubations in 400 or 500 ul HEPES buffer alone (basal). Then, the HEPES buffer was removed, saved for assay and replaced with HEPES buffer alone or HEPES buffer containing calcium channel blockers or serotonergic drugs (pretreatment). Likewise, 10 minute incubations with the addition of 50 mM KCl to the HEPES buffer were utilized to evoke neurotransmitter release in the presence or absence of drugs. After stimulation the cells were exposed to HEPES buffer alone again to re-establish basal release. The supernatants were collected following each 10 minute interval for CGRP radioimmunoassay and/or glutamate assay. Each drug and diluent utilized (calcium channel blockers, serotonergic drugs, pertussis toxin) was tested to determine that it did not interfere with either the CGRP or glutamate assay by adding the indicated concentrations to replicate points in the standard curve. For example, the final concentration of DMSO used in some experiments was 0.01% or less and did not alter basal or stimulated release of CGRP or glutamate or CGRP or glutamate assays (data not shown).

### CGRP radioimmunoassay

Total immunoreactive CGRP released into the buffer was determined by radioimmunoassay as previously described [58]. Briefly, 25 μl of CGRP antibody (1:130,000 dilution) and 25 μl of [^125^I] [Tyr^0^] CGRP (28–37) containing 4000–6000 cpm were added to each sample and to the standards. The samples were incubated with radiolabeled peptide and antiserum for 16 – 20 h at 4°C. Tracer not bound to antibody was removed from tracer bound to antibody by adding 0.5 mL of a 0.1 M phosphate buffer (pH 7.4) containing 1% Norite charcoal, 50 mM NaCl, and 0.1% bovine serum albumin. This mixture was centrifuged at 1000 × G for 20 min at 4°C, the supernatant was decanted, and the radioactivity was measured by gamma scintillation spectrometry. The amount of CGRP in unknown samples was estimated by comparing the percent bound radioactivity in unknowns to a standard curve using a four-point nonlinear least squares regression analysis. Using this method, the minimal detectable amount of CGRP was 0.5 fmol.

### Glutamate assays

Glutamate was measured by HPLC separation and electrochemical detection of the OPA-mercaptoethanol derivative using modifications of the methods of Donzanti & Yamamoto [59,60]. Using this method, the minimal detectable amount of glutamate was 2.5 pmol.

### Statistical analysis

Each experiment was completed at least three times with independent trigeminal preparations. In some experiments, we assayed both glutamate and CGRP from the same wells. Data from individual experiments were pooled for graphical presentation and further statistical analysis with GraphPad software (San Diego, CA). Inhibition of CGRP or glutamate release was calculated by comparing the release evoked by potassium in the presence of drug to the release evoked by potassium in the absence of drug and displayed as a percent for each experiment and averaged across experiments. Data are presented as mean ± S.E.M. Repeated measures ANOVA was used to compare groups and where appropriate posthoc Bonferroni tests were performed. The significance level for all tests was set at p < 0.05.

## Competing interests

The authors declare that they have no competing interests.

## Authors' contributions

YX performed the CGRP and glutamate release experiments. JAR designed and performed the glutamate release experiments. JHH designed the release experiments and prepared the manuscript. All authors read and approved the final manuscript.
